# A systematic approach to ultrasound-guided central venous catheter placement—desirable modifications

**DOI:** 10.1186/s13054-017-1877-9

**Published:** 2017-12-08

**Authors:** Ryszard Gawda, Tomasz Czarnik

**Affiliations:** Department of Anesthesiology and Critical Care, Opole University Hospital, Aleja Witosa 26, 45-401 Opole, Poland

See related research by Saugel et al., https://ccforum.biomedcentral.com/articles/10.1186/s13054-017-1814-y

With much interest we read the paper written by Saugel et al. [1] on a systemic approach to ultrasound-guided central vein catheterization. The article is comprehensive but some issues need discussing.

The authors claim that the tip of the needle can be constantly identified while the needle is approaching the vein both in short-axis and long-axis views. This is inaccurate. The only approach where it is possible to constantly visualize the tip of the needle is the in-plane technique. In the short-axis view, the needle is visible as a white dot, which also applies to the tip of the needle as with any part of the needle shaft [2, 3]. It means that the tip can be within the lumen of the vein or below the vessel when the second wall of the vein is punctured.

The scheme created by the authors is similar to the one we recently published [4]. Unfortunately, their proposed systematic approach ignores several crucial steps in the procedure. Firstly, the needle is not always visualized perfectly. Therefore, before puncturing the vein, you need to check if the needle has not been positioned over the adjacent artery. How this is done depends on the technique used. It is simple in the short-axis view, when both vessels are constantly presented on the screen, but in the long-axis view, only the vein is visualized; thus, the angle needs to be changed between the ultrasound probe and the skin in order to visualize the adjacent artery. When the tip of the needle is not centrally positioned over the vein, the needle can miss the vein, also damaging the adjacent artery.

The other important step is to test the introduction of the guidewire, i.e., to check whether the tip of the guidewire is not being introduced into the other central vein on the same side of the patient. This problem mostly concerns the jugular vein during axillary/subclavian vein catheterization and the axillary/subclavian vein during jugular vein catheterization. This step acts as a protection (but not completely) against introducing the catheter into an undesirable location.

In our opinion ultrasound-guided central venous catheter placement is a slightly more complex procedure than the one proposed in the paper. It not only involves introducing the catheter into the vein but also controls all the steps in order to guarantee the safety of the patient.
